# Quantitative proteomics identified circulating biomarkers in lung adenocarcinoma diagnosis

**DOI:** 10.1186/s12014-022-09381-x

**Published:** 2022-11-21

**Authors:** Hongyu Chen, Xiaoqin Lai, Yihan Zhu, Hong Huang, Lingyan Zeng, Li Zhang

**Affiliations:** 1 Key Laboratory of Transplantation Engineering and Immunology, Ministry of Health, West China Hospital, Sichuan University, Chengdu, Sichuan China; 2 Laboratory of Pathology, West China Hospital, Sichuan University, Chengdu, Sichuan China; 3 Institute of Respiratory Health, West China Hospital, Sichuan University, Chengdu, Sichuan China; 4 Day Surgery Center, West China Hospital, Sichuan University, Chengdu, Sichuan China; 5 Frontiers Science Center for Disease-Related Molecular Network, West China Hospital, Sichuan University, Chengdu, Sichuan China

**Keywords:** Lung adenocarcinoma, Proteomics, Biomarkers, Early diagnosis, UQCRC1, RAC1

## Abstract

**Background:**

Lung cancer (LC) is a common malignant tumor with a high incidence and poor prognosis. Early LC could be cured, but the 5-year-survival rate for patients advanced is extremely low. Early screening of tumor biomarkers through plasma could allow more LC to be detected at an early stage, leading to a earlier treatment and a better prognosis.

**Methods:**

This study was based on total proteomic analysis and parallel reaction monitoring validation of peripheral blood from 20 lung adenocarcinoma patients and 20 healthy individuals. Furthermore, differentially expressed proteins closely related to prognosis were analysed using Kaplan–Meier Plotter and receiver operating characteristic curve (ROC) curve analysis.

**Results:**

The candidate proteins GAPDH and RAC1 showed the highest connectivity with other differentially expressed proteins between the lung adenocarcinoma group and the healthy group using STRING. Kaplan–Meier Plotter analysis showed that lung adenocarcinoma patients with positive ATCR2, FHL1, RAB27B, and RAP1B expression had observably longer overall survival than patients with negative expression (*P* < 0.05). The high expression of ARPC2, PFKP, PNP, RAC1 was observably negatively correlated with prognosis (*P* < 0.05). 17 out of 27 proteins showed a high area under the curve (> 0.80) between the lung adenocarcinoma and healthy plasma groups. Among those proteins, UQCRC1 had an area under the curve of 0.960, and 5 proteins had an area under the curve from 0.90 to 0.95, suggesting that these hub proteins might have discriminatory potential in lung adenocarcinoma, *P* < 0.05.

**Conclusions:**

These findings provide UQCRC1, GAPDH, RAC1, PFKP have potential as novel biomarkers for the early screening of lung adenocarcinoma.

**Supplementary Information:**

The online version contains supplementary material available at 10.1186/s12014-022-09381-x.

## Introduction

Cancer remains the leading cause of death and poses a significant burden of disease in the world for both males and females. With an estimated 2,206,771 new cases and 1,796,144 deaths in 2020, lung cancer (LC) poses a serious threat to human health and life [1]. The incidence and mortality of LC in China are far higher than those in other countries. According to statistics in 2020 by GLOBOCAN, there were 815,563 LC patients in China, including 714,699 deaths. The total number of deaths from LC is greater than that of those who died of colon cancer, prostate cancer, and breast cancer [2]. Non-small cell lung cancer (NSCLC) is the most common LC, accounting for about 80% of cases. In which, lung adenocarcinoma (LUAD) is the most common type of NSCLC, making up 40% of LC [3]. LUAD occurs at a younger age and generally has no obvious symptoms in the early stage. The diagnosis of LC depends mainly on imaging examination (X-ray, CT scan, positron emission tomography, and magnetic resonance imaging) [2, 4, 5]. The high-risk individual guideline of the National Comprehensive Cancer Network suggests high-risk groups aged 50 or above and 20 pack-years of smoking history low-dose computed tomography for each year. However, these procedures are unsuitable for disease screening, let alone continuous surveillance. In comparison, peripheral blood examination is characterized by convenience, economy, and strong operability and has higher clinical application value. The early symptoms of LC are not obvious and are often not diagnosed until the later stages. The treatment of NSCLC is stage-specific. For patients with stage I or II disease, the best treatment is complete surgical resection, and the 5-year survival rate after surgery can reach more than 80% [6]. Most patients with advanced LC have metastases, and comprehensive treatments such as local radiotherapy, systemic palliative chemotherapy, targeted therapy, and traditional Chinese medicine can be selected to control the growth rate of tumors and prolong the survival time [7]. With the rapid development of molecular diagnosis, LC patients have ushered in a new approach to targeted immunotherapy. Finding new biomarkers to improve diagnostic accuracy will contribute to its better application in clinical practice.

Mass spectrometry (MS) technology is widely used to analyze proteomics and can detect proteins in biological materials with high throughput [8]. Multiple reaction monitoring is a classical targeted proteomic detection technology that requires the first locking of a parent ion and a daughter ion of a protein, followed by the collection of ion pairs (parent ion/daughter ion) by triple quadrupole mass spectrometry [9]. Parallel reaction monitoring (PRM), based on multiple reaction monitoring, locks in a single parent ion and subsequently detects all the daughter ions of the parent ion. PRM has higher selectivity, better sensitivity, better reproducibility, and better anti-interference ability in complex backgrounds [10–12]. Proteomics has a direct effect on human diseases, has been widely used in human diseases such as skin disease, cancer, and heart disease [13–16]. The research mainly focuses on searching for individual proteins related to diseases, studying the changes in protein expression or modification caused by certain diseases, and using the proteome to search for diagnostic markers and vaccines for diseases caused by pathogenic microorganisms [17, 18]. In recent years, omics studies of LC have mainly been in the classification of LC, development of LC, and exploration of new biological targets [5]. The relatively different protein abundance in healthy and disease samples was provided by PRM, which may provide sources for clinical validation studies and help us to understand the occurrence and prognosis of LC.

In this research, we conducted a comparative analysis of proteins collected from the peripheral blood of LUAD patients. We utilized LC–MS/MS technology for proteomic analysis of 10 LUAD plasmas and 10 healthy plasmas. We identified 317 differential expression proteins (DEPs). Then, 40 DEPs associated with prognosis were tested for PRM in another 10 plasma pairs of LUAD patients and healthy individuals. Using the KM-Plotter database, the overall survival (OS) and 10 genes with the highest differential expression of proteins in LUAD patients were studied. It is suggested that FHL-1, RAB27B, and RAP1B may be biomarkers for the good prognosis of LUAD, while ARPC2, PFKP, RAC1, and PNP may be biomarkers for malignant prognosis of LUAD. The diagnostic potential of the top 10 DEPs was calculated by receiver operating characteristic curve (ROC) curves. 17 proteins had high diagnostic potential, whose area under the curve (AUC) > 0.80, and the AUC of CCT7 reached 0.960 especially.

## Methods

### Clinical sample collection

This retrospective study was approved by the Research Ethics Committee of West China Hospital of Sichuan University. This study involved 40 plasma samples, including 20 from patients with lung adenocarcinoma and 20 from healthy individuals (Table 1). The 40 samples were randomly divided into two groups, each consisting of plasma from 10 patients with lung adenocarcinoma and 10 healthy subjects, for mass spectrometry and PRM analysis. Most of these samples (> 70%) were from patients with early lung adenocarcinoma in the LUAD group, and more than 80% had no history of smoking. Information gathered from the patient's medical records and follow-up included specimen collection time, sex, diagnosis age, admission time, discharge diagnosis, pathology, smoking history, surgical method, Tumor Node Metastasis stage, pathological report, and routine blood test.

**Table 1:** Clinical information of patients included in LC–MS/MS and PRM

Variable	LC–MS/MS		PRM	
	LUAD (n = 10)	Control (n = 10)	LUAD (n = 10)	Control (n = 10)
Age (year)	58.0 ± 12.3	44.4 ± 13.8	55.8 ± 8.5	44.5 ± 14.1
Gender; n (%)				
Male	3 (30%)	7 (70%)	3 (30%)	6 (60%)
Female	7 (70%)	3 (30%)	7 (70%)	4 (40%)
Stage; n (%)				
I	8 (80%)		7 (70%)	
II	2 (20%)		1 (10%)	
III			2 (20%)	
IV				

### Sample preparation

The lysis buffer was mixed with the sample and was cleaved by ultrasound. After centrifugation, the supernatant was retained and the protein was precipitated in 20% TCA and precooled acetone solution. The protein precipitate was dissolved in urea solution and the concentration extracted from every sample was determined by the BCA kit following the manufacturer’s instructions. The lysate was added to the sample and then dithiothreitol was added and incubated at 56 ℃ for 30 min. Iodoacetamide was added and incubated. After cleaning with urea and buffer solution 3 times, trypsin was added overnight. The peptide was centrifuged at 12000*g* for 10 min to recover.

### PRM analysis based on LC–MS/MS

Based on the proteomic analysis, we only considered the role of prognostic proteins that differed between lung adenocarcinoma and the healthy group 40 proteins were screened from DEPs to conduct the next PRM analysis. An ULTRA-performance liquid phase system was used to isolate peptides, and ionization analysis was performed using Orbitrap Exploris 480. High-resolution Orbitrap was used to detect and analyze peptide parent ions and their secondary fragments.

### Proteomic data analysis

After raw files from MS detection, quality control, and analysis of raw data according to database retrieval results. Secondary MS data of this experiment were searched by Proteome Discoverer (V2.4.1.15). The database was homo_sapiens_9606_PR_20201214. Fast A (75777 sequences), and figured up the false discovery rates (FDR) by random matching. The number of missing tangent positions was set as two, the minimum peptide length was set as six, and the maximum modification number of peptides was set as 3. The FDRs were set at 1% in the whole process.

### Identification of DEPs

The DEPs in the LUAD group compared with the NL group were calculated as follows, where R represents relative protein quantity and P represents protein: FC_LUAD/NL, P_ = Mean (R_LUAD, P_)/Mean (R_NL, P_). To amplify protein differences, FC needs to undergo log base 2 conversions. After the above difference analysis, when the *P* value of *t*-test ≤ 0.05, Log_2_FC > 1.5 were up-regulated DEPs, and Log_2_FC < 1/1.5 were down-regulated DEPs.

### GO/KEGG analysis

In proteomics projects, Gene Ontology (GO) annotations for proteins are split into biological processes, cell composition, and molecular function. The *P* value of Fisher’s exact test ≤ 0.05. The Kyoto Encyclopedia of Genes and Genomes (KEGG) database is an information network linking known interactions between molecules. When the *P* value of Fisher’s exact test ≤ 0.05, the pathway enrichment was considered significant.

### PPI analysis

Protein–protein interaction (PPI) is used to analyze the interactions between proteins, including direct physical interactions and indirect functional correlations. 27 DEPs from PRM in LUAD groups compared with NL group were analyzed with the protein network interaction database of STRING (V.11.5, https://cn.string-db.org/). Line color indicates the type of interaction evidence: the light blue indicates from curated Databases, the pink indicates experimentally determined, the green indicates gene neighborhood, the red indicates gene fusion, the dark blue indicates gene co-occurrence, the yellow indicates text mining, the dark indicates co-expression, the purple indicates protein homology.

### Kaplan–Meier plotter analysis

KM-Plotter is a publicly available online survival analysis site based on data from GEO, EGA, and TCGA [19]. At present, more than 54,000 gene expression and related survival data of 21 cancers have been collected, including 3452 LC patients, and relevant data were for the analysis of DEPs on LUAD. In the KM-Plotter online analysis site, the patient’ sample was divided into two groups by inputting 10 genes, using the best-performing threshold between the upper and lower quartiles as a cutoff value. Overall survival (OS) analysis was performed to obtain a survival map containing risk factors, *P* < 0.05 was a requirement.

### ROC analysis

ROC analysis was performed on targeted proteomic quantitative proteins to assess their sensitivity and specificity by MedCalc Statistical Software version 19.0.4. The AUC was used to estimate the diagnostic value. A logistic regression model was constructed for protein combination analysis.

### Western blot

We used western blot (WB) experiment to verify the PRM results. Plasma samples were quantified by the Enhanced BCA Protein Assay Kit (Beyotime), diluted to 5 µg/µl by 6X SDS-PAGE Sample Loading Buffer (Biosharp) and ddH_2_O, heated at 100℃ for 10 min. 50 µg protein per sample were electrophoresed in 10% polyacrylamide gels and transferred to 0.2 μm PVDF membranes (Bio-rad). The membrane was stained and quantified in Ponceau S (Servicebio), then cleaned and blocked in TBST containing 5% skim milk powder for 1 h. Then it incubated at 4 ℃ with primary antibody overnight, with secondary antibody at room temperature for 2 h, and an electro chemiluminescent reagent was used for chemiluminescence detection. The primary antibodies were CCT7 (Proteintech), UQCRC1 (Proteintech), PGD (Proteintech), LDHA (Proteintech), GAPDH (PTM-bio), and GP5 (Santa Cruz Biotechnology).

### Immunohistochemistry

Immunohistochemistry (IHC) was used to supplement the PRM results at the tissue level. Paraffin sections were dewaxed, antigen repaired, incubated with 3% hydrogen peroxide, and added with primary antibody at 4 ℃ overnight and anti-mouse/rabbit secondary antibody (Dako) at room temperature for 1 h. After DAB chromogenic agent (Dako) and hematoxylin were used, the slides were dehydrated and sealed. The primary antibodies were the same as WB’s.

## Results

### Global proteome characterization of LUAD and healthy plasma

**Fig. 1:**
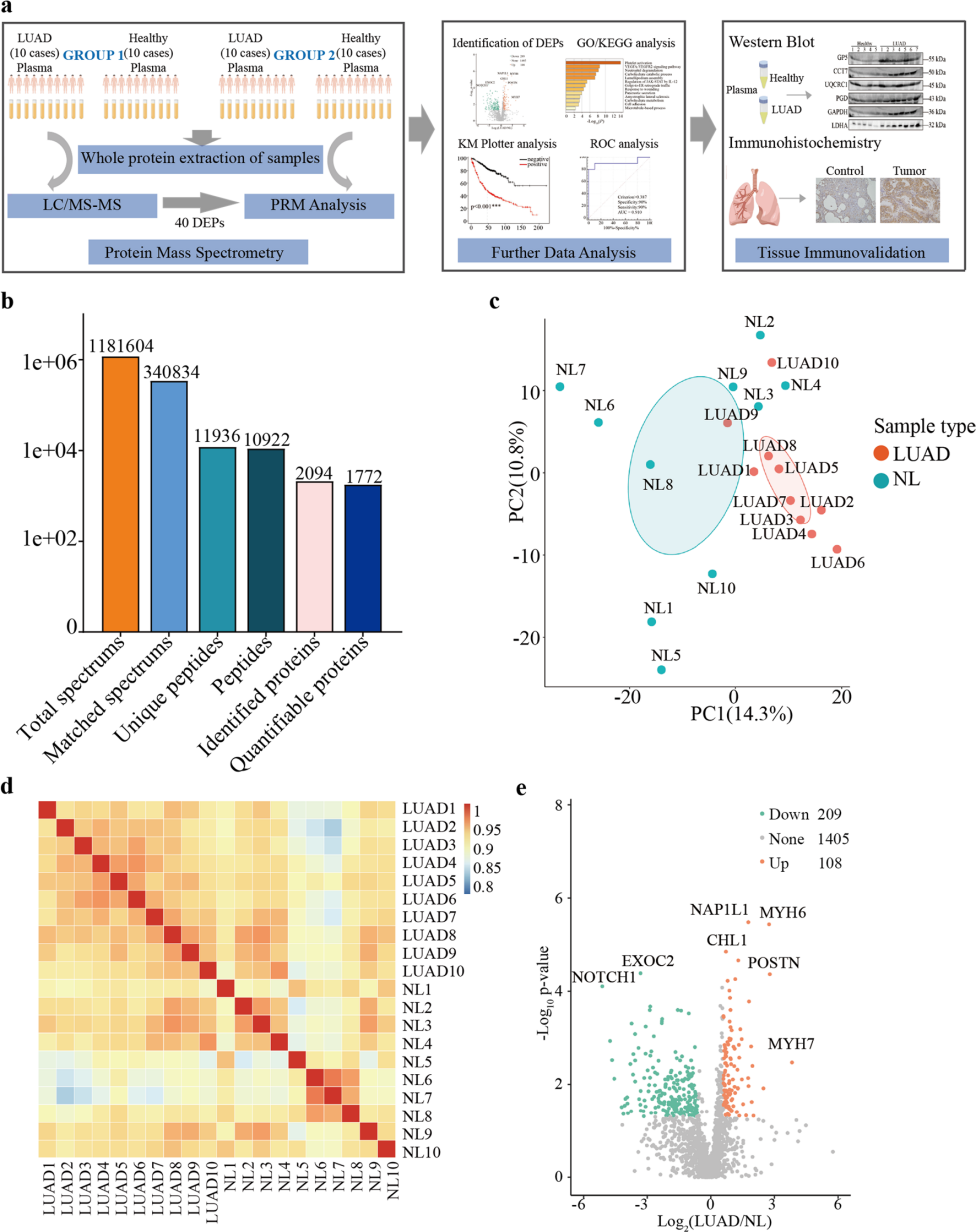
Global proteome characterization of LUAD and healthy plasma **a** The protocol of detailing experiments **b** Identification of quantifiable proteins from MS/MS spectra **c** Principal Component Analysis of quantified proteins, a complete separation of LUAD and NL groups **d** Pearson’s Correlation Coefficient of LUAD and NL groups. **e** volcano plot of all proteins in LUAD compared to NL

The mass and signal intensity of peptide and fragment ions after peptide fragmentation were obtained by mass spectrometry. The information at the peptide level is called the primary spectrum, and the information at the peptide fragment ion is called the secondary spectrum. Secondary MS data were retrieved by Proteome Discoverer (V2.4.1.15), Homo_sapiens_9606_PR_20201214. fasta database. In this research, proteomic analysis was matched on 10 LUAD peripheral blood (7 female and 3 male) and 10 healthy peripheral blood (Fig. 1a). A total of 1,181,604 secondary mass spectra were collected (Fig. 1b). 340,834 spectra are matching theoretical secondary spectra in the database, with a utilization ratio of 28.85%. A total of 11936 peptide sequences were identified from the matching results, including 10922 unique peptide sequences. During quantification, one protein corresponded to multiple specific peptides, and 2094 identification proteins were identified by specific peptides, resulting in 1772 proteins. Principal component analysis was used to show a general pattern of changes in protein abundance within and between the two groups to observe the similarities and differences between samples. As shown in Fig. 1c, the LUAD group exhibited clustering specificity, while the healthy group spread out randomly. The Pearson correlation coefficient between samples is shown by a heatmap. The correlation coefficient between proteomics data is shown in Fig. 1d, and the correlation between LUAD samples was 0.916–0.947 and between healthy samples ranged from 0.890 to 0.945, indicating a significant correlation.

### Proteomic features of DEPs in LUAD

**Fig. 2:**
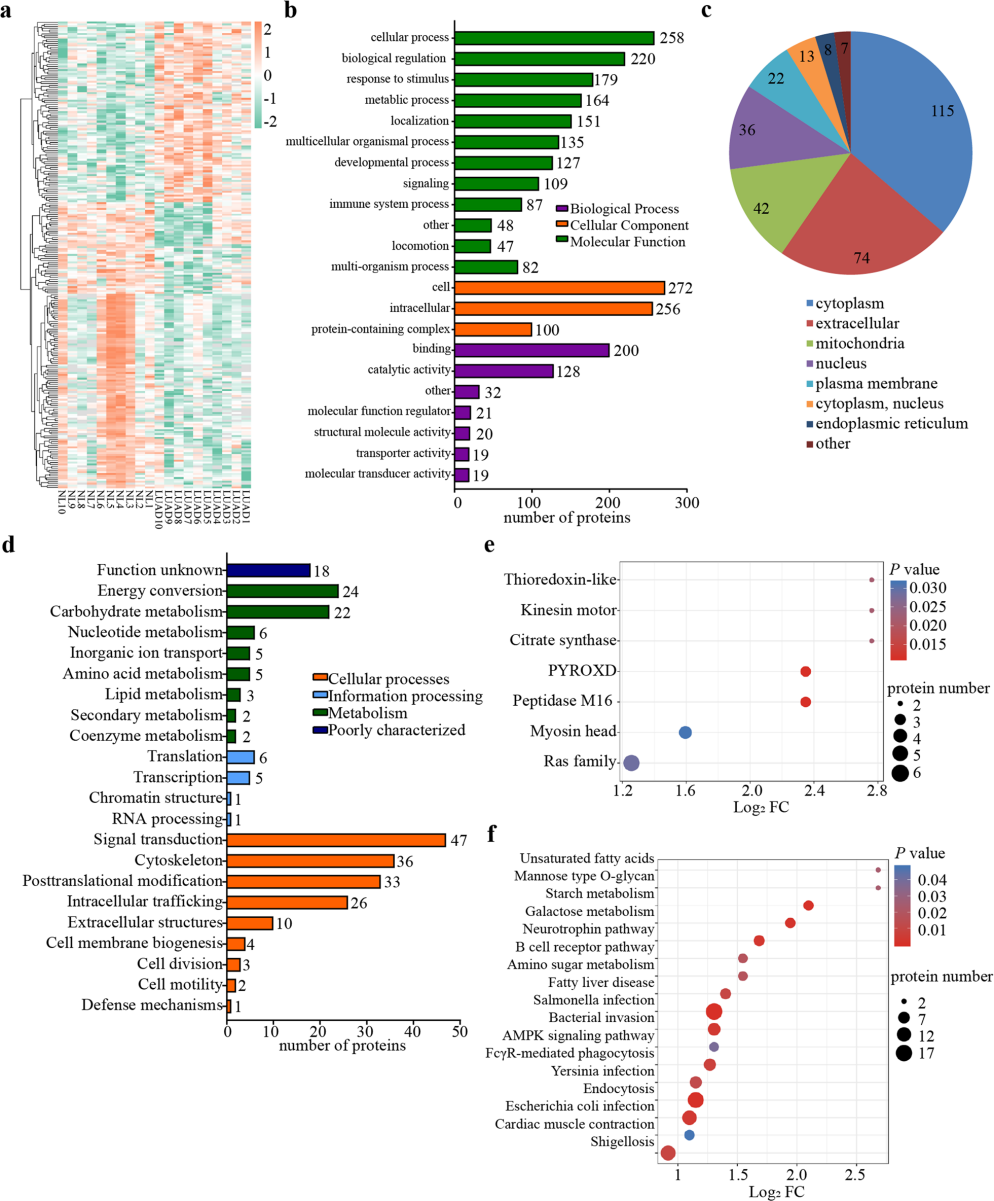
Proteomic features of differentially expressed proteins in lung adenocarcinoma **a** Heatmap with hierarchical clustering of DEPs **b** The orange indicating high expression and green indicating low expression. Each line represents a protein, and each column represents a sample **c** GO analysis of DEPs. **d** subcellular localization prediction of DEPs **e** KOG categories of DEPs **f** KEGG enrichment analyses of DEPs

To judge the conspicuousness of the difference in protein expression, we performed a *t*-test on the Log_2_FC of each protein in the LUAD group compared with the NL group. When the *P* value was ≤ 0.05, Log_2_FC > 1.5 were up-regulated DEPs, and Log_2_FC < 1/1.5 were down-regulated DEPs, and 317 DEPs were finally obtained. Compared with healthy controls, in the quantifiable proteins, there are 208 up-regulated proteins and 109 down-regulated proteins in the LUAD group signally, like MYH6, POSTN, NAP1L1, EXOC2 and NOTCH1. A volcano plot was drawn exhibiting DEPs in statistics in LUAD in comparison with the NL group (Fig. 1e). Furthermore, the heatmap also represented a hierarchical cluster of the DEPs (Fig. 2a). According to Gene Ontology (GO) functional classification for the 317 DEPs, they were cataloged into three categories and 22 terms, including 12 biological processes, three cellular components, and seven molecular functions (Fig. 2b). These proteins are involved in cellular processes, binding and catalytic activity. Figure 2c shows that about 36% DEPs were in the cytoplasm (115 proteins), 23% in extracellular space (74 proteins), 13% in mitochondria (42 proteins), and 11% in the nucleus (36 proteins), which suggests that DEPs of LUAD may be secreted to carry out signal transduction, participate in tumor energy metabolism through mitochondria, and regulate gene expression in the nucleus. The Clusters of Orthologous Groups of proteins (COG) categories indicated that the DEPs were related to energy metabolism, carbohydrate metabolism, signal pathway and mechanisms, cytoskeleton and protein modification after translation, protein transportation and chaperones (Fig. 2d).

### KEGG and GO enrichment analyses of DEPs in plasma

To further predict the possible roles of DEPs, we conducted functional enrichment analysis using Fisher’s exact test, *P* < 0.05 is required. The GO analysis included biological processes, cellular component, and molecular function annotations, explaining the function of DEPs in multiple angles. The upregulated proteins in the plasma of LUAD patients compared with healthy subjects were significantly enriched in neurogenesis, negative regulation of cell communication and signal transduction, positive regulation of cell death and other biological processes (Fig. 2e). According to the cellular component annotation, the majority of the DEPs originated from the endoplasmic reticulum, extracellular space, Golgi apparatus, and endoplasmic reticulum lumen and are involved in protein processing and transport. The molecular function analysis revealed that DEPs were enriched in ionic binding activity and protein heterodimerization activity (Fig. 2f), which act a pivotal part in transmembrane transports and biological information transfer. The downregulated proteins were centered on phosphorylation, regulation of organelle organization, ribose and nucleoside phosphate metabolic processes. Cellular component annotation was focused on the cytoskeleton, mitochondrion, microtubule cytoskeleton, and other regions related to cell proliferation. The molecular functions of the downregulated proteins were enriched in anion binding, small molecule binding and nucleotide binding, which is consistent with the upregulated proteins.

### Development of targeted protein assays using PRM

**Fig. 3:**
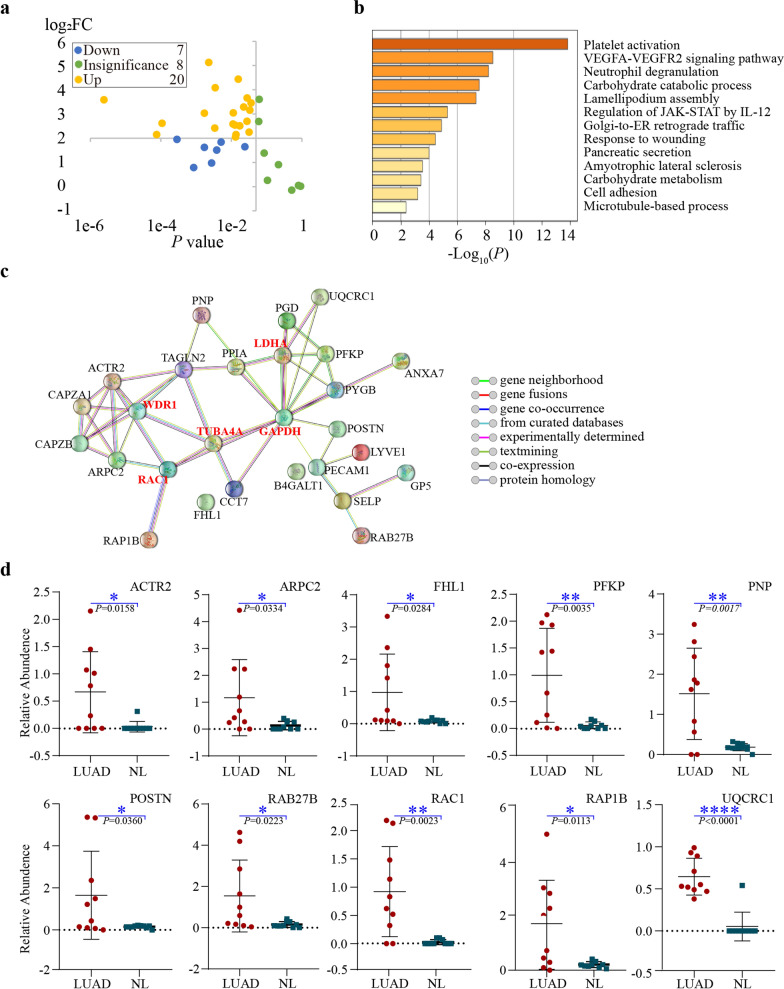
Detection of targeted proteins by PRM **a** 35 proteins according to the adjusted *P* value are colored in blue (down-regulated) and yellow (up-regulated) **b** GO analysis of 27 DEPs detection by PRM **c** PPI network analysis was performed using the STRING11.0 online software. **d** the graph demonstrates individually the top 10 significant DEPs, *P* value < 0.05

To further extend the research for application and prognosis role, PRM detection was further tested. The 317 DEPs obtained previously were retrieved from the database, and the next study focused on 40 proteins closely related to prognosis. PRM testing was performed in peripheral blood from an additional 10 LUAD patients and 10 healthy subjects, of which 35 proteins were quantitatively analyzed, limited by certain protein characteristics and expression abundance. 27 proteins were statistically significant, including 20 up-regulated differential proteins and seven down-regulated DEPs (Additional file 1: Table S1). The obtained data were processed by Skyline (v.3.6) to calculate protein relative abundance (Fig. 3a). The DEPs were involved in platelet activation, VEGFA-VEGFR2 signaling pathway, and carbohydrate catabolic process (Fig. 3b).

As illustrated in Fig. 3D, we researched the altered proteins in STRING (V.11.5) and accessed the PPI network (Fig. 3c); these proteins may have a specific role in the early occurrence of LUAD. GAPDH, TUBA4A, WDR1, and LDHA are the central proteins shown in the network. The candidate proteins GAPDH and RAC1 showed the highest connectivity with other differentially expressed proteins between LUAD and NL using STRING by calculating the ratio of LUAD/NL, the expression levels of the top 10 significant DEPs, RAC1, ACTR2, PFKP, FHL1, UQCRC1, POSTN, RAB27B, ARPC2, RAP1B, and PNP, are shown in Fig. 3d.

**Fig. 4:**
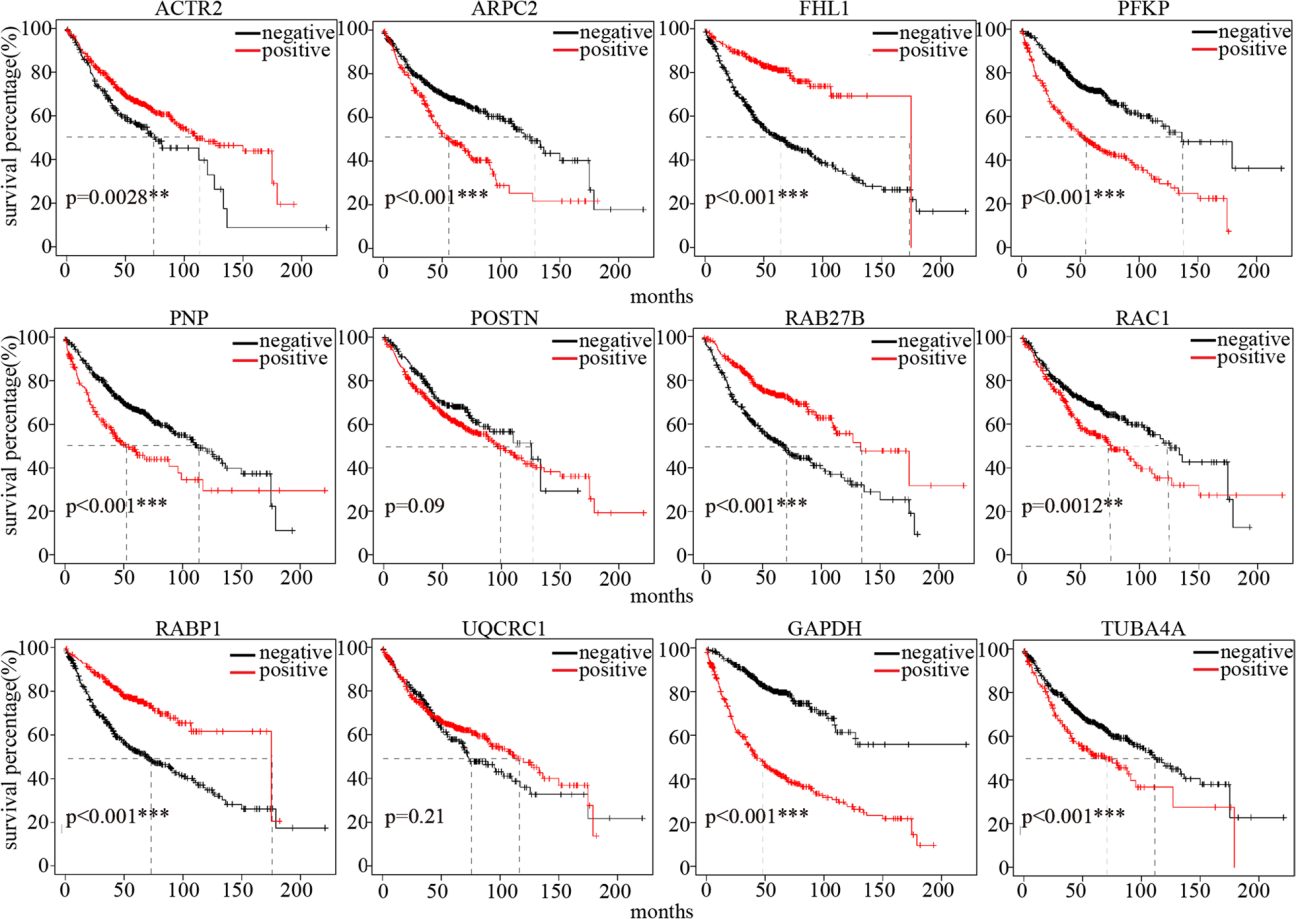
Kaplan–Meier survival analysis of LUAD group compared with NL group

KM-plotter was used to evaluate the prognosis performance of every DEP. The KM analysis showed that LUAD patients with positive ATCR2, FHL1, RAB27B, and RAP1B (Fig. 4) expression had observably longer OS than patients with negative expression (*P* < 0.05). The high expression of ARPC2, PFKP, PNP, RAC1, GAPDH, and TUBA4A was observably negatively correlated with OS prognosis (*P* < 0.05). ARPC2 is involved in the control of intracellular dynamic changes of actin and facilitates cell migration and tumor metastasis in lung, colon, and breast cancer. PFKP is a platelet-specific phosphofructokinase that makes a critical difference in metabolic reprogramming in certain cancers, including bladder cancer, breast cancer, and lung cancer, and is a potential driver gene in the GEO (Gene Expression Omnibus) database [20–24]. RAC1 (Rac family small GTPase 1) is a driver gene that regulates plenty of cellular events, particularly in cell growth and division, cytoskeletal and synaptic recombination, autophagy, and tumor metastasis. These 10 genes were analyzed for combined KM prognosis (Fig. 4) [25–27].

Overall survival of ATCR2, FHL1, RAB27B, RAP1B, UQCRC1, ARPC2, PFKP, PNP, POSTN, RAC1, GAPDH and TUBA4A were performed in KM-Plotter online survival analysis site

### Potential diagnostic markers in LUAD

**Fig. 5:**
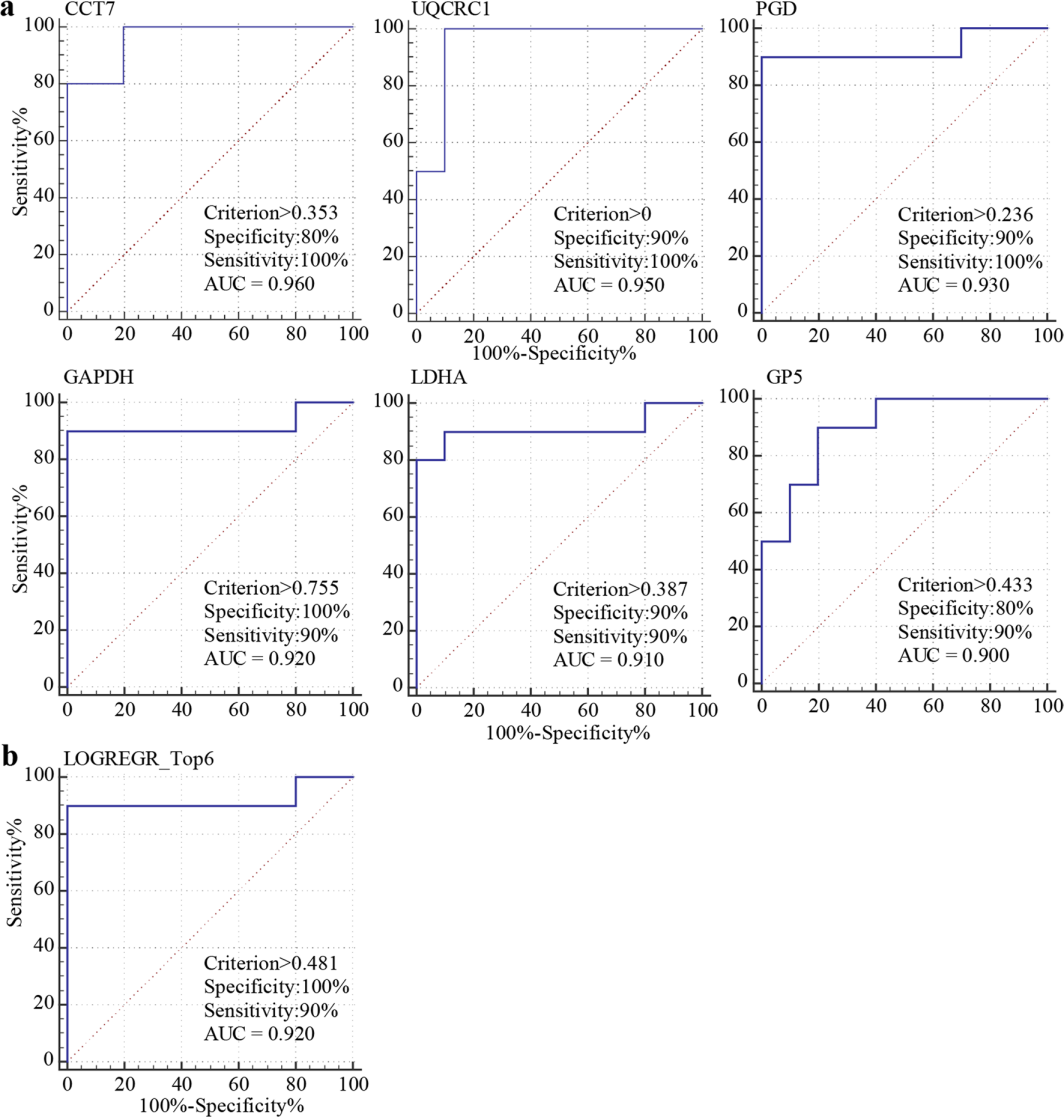
ROC curves of the best protein **a** The AUC values of six proteins in these datasets **b** The AUC values of the combination of six-protein

The samples used in this study were mainly concentrated in early LUAD, so the next step was to draw ROC curves to check the effect of each DEPs in the diagnosis of LUAD. If the AUC was > 0.70, the proteins could be regarded as a potential independent diagnostic factor. The mean plasma protein expression of 10 healthy people was negative, and ROC analysis was performed on the 27 proteins with differential expression by Medcalc software. 17 out of 27 proteins revealed a high AUC (> 0.80) between the LUAD group and NL group. Among those proteins, CCT7 had an AUC of 0.960, and there were five proteins with an AUC from 0.90 to 0.95, indicating that these central proteins might have the discriminative capacity in LUAD (Fig. 5a), *P* < 0.05. Logistic regression analysis was performed for 6 plasma proteins with higher AUCs, including CCT7, UQCRC1, PGD, GAPDH, LDHA, and GP5 (Table 2), resulting in a detection rate of 92% (Fig. 5b). UQCRC1 acts on cytochrome C upstream or internally of mitochondrial electron transport and has been studied extensively in Alzheimer's disease, which may play an important role in the targeted therapy of pancreatic cancer [28–30].

**Table 2:** Six differentially expressed proteins identified in plasma samples

No	Uniprot accession	Gene	Protein name	Protein function
1	P31930	UQCRC1	Ubiquinol-cytochrome c reductase core protein 1	Enzymes, predicted intracellular proteins, Metallopeptidases
2	Q99832	CCT7	Chaperonin containing TCP1 subunit 7	Predicted intracellular proteins
3	P52209	PGD	6-Phosphogluconate dehydrogenase	Enzymes, predicted intracellular proteins, Oxidoreductases
4	P04406	GAPDH	Glyceraldehyde-3-phosphate dehydrogenase	Enzymes, predicted intracellular proteins, Oxidoreductases
5	P00338	LDHA	Lactate dehydrogenase A	Candidate cancer biomarkers, Enzymes, Oxidoreductases, Predicted intracellular proteins
6	P40197	GP5	Glycoprotein V platelet	Hemostatic, CD markers

### Experimental verification by western blot and immunohistochemistry

**Fig. 6:**
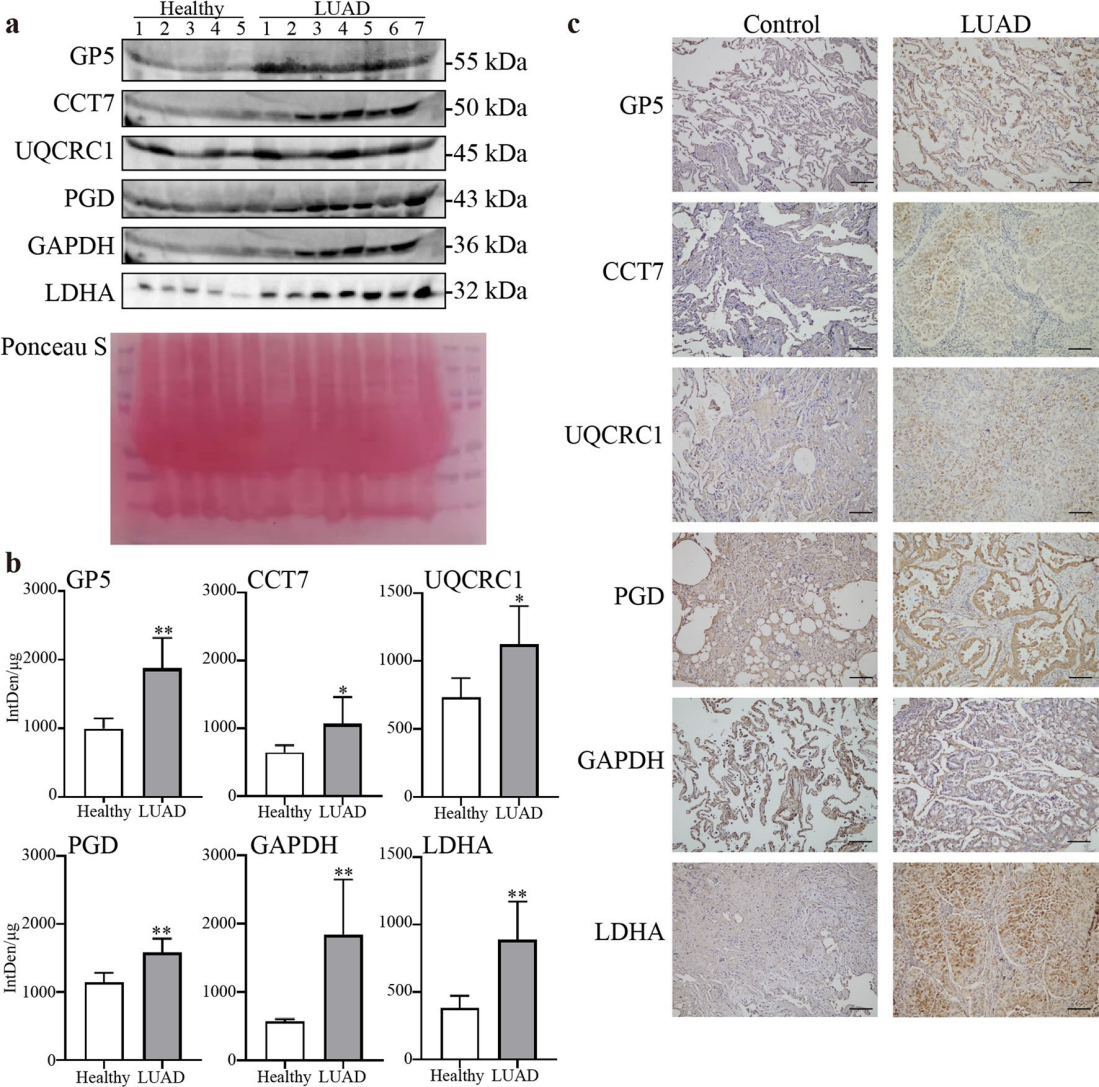
Western blot analysis and immunohistochemistry staining of six proteins **a** Western blot of GP5, CCT7, UQCRC1, PGD, GAPDH, LDHA in healthy and LUAD plasmas, Ponceau S was used for quantification of total protein, **b** histogram of protein expression level calculated by line’s IntDen divide total protein per lane (µg). **c** Immunohistochemistry staining of GP5, CCT7, UQCRC1, PGD, GAPDH and LDHA in LUAD tissues and tumor-adjacent tissues; scale bar = 100 µm (magnification, × 200). (*P value < 0.05, **P value < 0.01)

We examined the expression of these 6 potential biomarkers by WB detection in 25 plasma samples (14 patients and 10 controls) and immunohistochemical detection in 22 lung tissues (10 LUAD and 12 tumor-adjacent tissues) (Table 3). The expression of GP5, CCT7, UQCRC1, PGD, GAPDH and LDHA were higher in the LUAD patients’ plasma (Fig. 6a, b), which basically consistent with the PRM’s result. In addition, we also verified the expression of those proteins in LUAD tissues and tumor-adjacent tissues by IHC with a similar trend (Fig. 6c).

**Table 3:** Clinical information of patients included in WB and IHC

Variable	WB		IHC	
	LUAD (n = 14)	Control (n = 10)	LUAD (n = 10)	Control (n = 12)
Age (year)	65.6 ± 8.7	83.5 ± 9.4	59.7 ± 7.1	58.2 ± 13.4
Gender; n (%)				
Male	5 (36%)	5 (50%)	5 (50%)	6 (50%)
Female	9 (64%)	5 (50%)	5 (50%)	6 (50%)
Stage; n (%)				
I	11 (78%)		4 (40%)	2 (16.7%)
II	3 (22%)		2 (20%)	5 (41.7%)
III			2 (20%)	4 (33.3%)
IV			2 (20%)	1 (8.3%)

## Discussion

Lung adenocarcinoma is a common malignant tumor with a high incidence and poor prognosis. However, the early symptoms of LUAD are not obvious, and diagnosis is difficult. The delay in the diagnosis of lung adenocarcinoma causes plenty of patients with malignant tumors to lose the opportunity for early treatment [6]. Compared with the imaging and histological methods commonly used for the early diagnosis of LC, blood tests have the advantages of low cost, convenience, and repeatable sampling and have a great influence on the diagnosis, treatment, and prognosis of tumors [4, 31]. The results of this research are on the strength of total proteomic analysis and PRM validation of peripheral blood from 20 LUAD patients and 20 healthy individuals, which is a source of several biomarkers with clinical application. Subsequent GO/KEGG analysis showed that the DEPs were mostly located in the endoplasmic reticulum, extracellular space, and Golgi apparatus, which take an active part in protein processing and transport and are enriched in substance transport, information transfer, and the cytoskeleton. Furthermore, 40 proteins closely related to prognosis were detected by PRM, and 10 of the most significant DEPs, RAC1, ACTR2, PFKP, FHL1, UQCRC1, POSTN, RAB27B, ARPC2, RAP1B, and PNP, were obtained.

In recent years, biomarkers of LC have become a research focus, and biomarker detection has shown a certain potential in LC screening. Combining biomarkers, imaging omics, and artificial intelligence to establish a comprehensive model for LC screening prediction may be the development direction of improving LC screening ability in the future [32–34]. Chapman et al. detected 7 autoantibodies [p53, C-Myc, and hEGF, HER2, NY-ESO-1, CAGE, Mucin 1 (MUC1), and GBU4-5 antibodies] in 154 patients (104 LC patients and 50 normal), which had a diagnose sensitivity of 61% and specificity of 90%, respectively [35]. LC activates the complement cascade effect through the classical complement pathway, which downstream cleaves complement fragment 4D in increased concentrations in the body fluids of LC patients [36, 37]. The value of circulating tumor DNA (ctDNA) in guiding the precision therapy of advanced tumors has been confirmed by relevant studies, but its role in the diagnosis of early LC remains uncertain. Circulating tumor cells (CTCs) are tumor cells detached by a primary tumor or metastatic tumor located in peripheral blood [37, 38]. The number of CTCs is small and heterogeneous, so detection technology is required and being explored. Exosomes are considered as non-invasive or minimally invasive biomarkers with the potential of cancer detection. In addition, there are certain studies on exosomes as cancer immunotherapy in anticancer vaccines, reducing tumor exosomes to prevent adverse prognosis and exosomes as drug delivery carriers [39, 40]. Currently, tumor markers commonly used in the clinic, such as CEA, FRT, NSE, CYFRA21-1, CA50, SCC, and CA125, show increasing positive expression in advanced stages but have low sensitivity and specificity. Screening is aimed at high-risk healthy populations. The use of screening methods must be highly sensitive and specific to precancerous or very early cancer, and accuracy requirements are very high. The combined detection of autoantibodies is helpful for the early diagnosis of LC, but its sensitivity does not meet the needs of early screening of LC. Research on ctDNA is still in the early stage, and the size and release mechanism of ctDNA remain unclear. The sensitivity and specificity of existing detection techniques for ctDNA detection are not ideal [41]. The content of CTCs in peripheral blood is very low, with only a few CTCs in 1 billion blood cells, and the sensitivity and specificity of early LC diagnosis are not high.

Some of the DEPs we found did not appear in previous studies, which may be influenced by sample differences, different platforms, and screening criteria. Hence, more accurate methods of analysis and more samples are needed to confirm our findings. Ubiquinol-cytochrome c reductase core protein 1 (UQCRC1), a key component of mitochondrial complex III, plays an important role in mitochondrial metabolism. UQCRC1 has a carcinogenic effect in pancreatic ductal adenocarcinoma (PDAC) and can be used as a potential prognostic marker and therapeutic target for PDAC [30]. GAPDH is widely used as a standardized reference for WB and other experiments for its constant expression in the same cells or tissues generally. Several studies have linked GAPDH expression to liver cancer and T cell lymphoma [42–45]. This study found that GAPDH was different expressed in the plasma of LUAD and healthy people by PRM detection and WB as well. CCT7 has prognostic value in endometrial cancer, hepatocellular carcinoma and breast cancer [46]. GP5, also known as CD42d, is mainly expressed in platelets and megakaryocytes, involved in platelet adhesion and aggregation [47, 48]. LDHA is abnormally highly expressed in many cancers, including lung cancer, and is associated with malignant progression, is a biomarker for cancer diagnosis and prognosis [49]. PGD has been reported to promote hepatocellular carcinoma and prostate cancer through the AMPK pathway. In conclusion, GP5, CCT7, UQCRC1, PGD, GAPDH and LDHA have been found to play a role in tumor development, prognosis and even diagnosis in previous studies. GP5, a part of the receptor for von Willebrand factor, has been poorly studied in tumor and disease diagnosis. This is an interesting question as to why GP5 expresses stably different between LUAD and healthy people. RAC1 is an important intracellular signal transduction molecule that is closely related to the occurrence and development of malignant tumors and a tumor driver gene. RAC1 inhibitors improve resistance to the EGFR inhibitor gefitinib in LC patients [50]. The DNA methylation of PFKP (phosphofructokinase platelet) was significantly upregulated in tumors, and the detection of hepatocellular carcinoma was highly accurate [51].

The current study has some limitations. Firstly, the sample quantity used in this discovery phase was insufficient. The goal of the next step is to evaluate the action of these specific proteins in more samples. The role of these markers in the early stage of LC still needs to be explored. In addition, the next step is to combine biomarker detection and autoantibody detection in peripheral blood to increase the accuracy and specificity of early screening. In our study, the four protein signatures we narrowed had high diagnostic power between LUAD and NL, suggesting that they have potential as novel biomarkers for the early screening of LUAD. The study adds to our understanding of potential biomarkers for LUAD, providing new and specific therapeutic targets for this cancer (Additional file 1).

## Conclusion

In this study, 10 DEPs, RAC1, ACTR2, PFKP, FHL1, UQCRC1, POSTN, RAB27B, ARPC2, RAP1B, and PNP, were found to be associated with the prognosis of LUAD. Among the 27 DEPs, 17 proteins in the LUAD group and NL group had higher AUC (> 80). Among these proteins, CCT7 had an AUC of 0.960, and 5 of them had an AUC between 0.90 and 0.95, suggesting that these central proteins might have the discrimination ability of LUAD.

## Supplementary Information

**Additional file 1: Table S1.** Differentially expressed proteins identified in plasma samples.

## Data Availability

The datasets used and/or analysed during the current study are available from the corresponding author on reasonable request.

## Abbreviations

LC: Lung cancer
NSCLC: Non-small cell lung cancer
LUAD: Lung adenocarcinoma
MS: Mass spectrometry
PRM: Parallel reaction monitoring
DEP: Differential expression protein
KM-Plotter: Kaplan–Meier plotter
OS: Overall survival
WB: Western blot
IHC: Immunohistochemistry
FHL-1: Four and a half LIM domain 1
RAB27B: Member RAS oncogene family
RAP1B: Member RAS oncogene family
ARPC2: Actin related protein 2/3 complex subunit 2
PFKP: Phosphofructokinase, platelet
RAC1: Rac family small GTPase 1
PNP: Purine nucleoside phosphorylase
ROC: Receiver operating characteristic
AUC: Area under the curve
CCT7: Chaperonin containing TCP1 subunit 7
TNM: Tumor node metastasis
TCA: Trichloroacetic acid
BCA: Bicinchoninic acid
LC–MS/MS: Liquid chromatography tandem mass spectrometry
MS: Mass spectrometry
FDR: False discovery rates
NL: Normal
FC: Fold change
GO: Gene ontology
KEGG: Kyoto encyclopedia of genes and genomes
PPI: Protein–protein interaction
MYH6: Myosin heavy chain 6
POSTN: Periostin
NAP1L1: Nucleosome assembly protein 1 like 1
EXOC2: Exocyst complex component 2
NOTCH1: Notch receptor 1
COG: Clusters of orthologous groups
GAPDH: Glyceraldehyde-3-phosphate dehydrogenase
TUBA4A: Tubulin alpha 4a
WDR1: WD repeat domain 1
LDHA: Lactate dehydrogenase A
ACTR2: Actin related protein 2
UQCRC1: Ubiquinol-Cytochrome C reductase core protein 1
PNP: Purine nucleoside phosphorylase
APRC2: Actin related protein 2/3 complex subunit 2
PGD: Phosphogluconate dehydrogenase
GP5: Glycoprotein V platelet
C-Myc: MYC proto-oncogene
Hegf: Basic fibroblast growth factor
HER2: Human epidermal growth factor receptor 2
NY-ESO-1: New York esophageal squamous carcinoma antigen 1
CAGE: Cancer-associated antigen
MUC1: Mucin 1
CTC: Circulating tumor cell
ctDNA: Circulating tumor DNA
