# Supplementary material for: Quantitative proteomics identified circulating biomarkers in lung adenocarcinoma diagnosis
Source: Clin Proteomics. 2022 Nov 21;19:44. doi: 10.1186/s12014-022-09381-x (PMC9677906; doi:10.1186/s12014-022-09381-x)
Supplement: Supplementary file 1 — Additional file 1: Table S1. Differentially expressed proteins identified in plasma samples. [file 12014_2022_9381_MOESM1_ESM.docx]

Table S1. Differentially expressed proteins identified in plasma samples.

| **No.** | **UniProt Accession** | **Gene** | **Protein Name** | **Unique Peptides** | **P value** | **Fold Change**  **(LUAD/NL)** | |
| --- | --- | --- | --- | --- | --- | --- | --- |
| **1** | P31930 | UQCRC1 | Ubiquinol-cytochrome c reductase core protein 1 | STLNEIYFGK  IAEVDASVVR | 2.43E-06 | 12.04212 | |
| **2** | Q99832 | CCT7 | Chaperonin containing TCP1 subunit 7 | LLALGDSGVGK  TATQLAVNK | 7.57E-05 | 4.461211 | |
| **3** | P52209 | PGD | Phosphogluconate dehydrogenase | IIEETLALK  AGQAVDDFIEK | 0.000108 | 6.157982 | |
| **4** | P04406 | GAPDH | Glyceraldehyde-3-phosphate dehydrogenase | VFASLPQVER | 0.000287 | 3.881287 | |
| **5** | P16284 | PECAM1 | Platelet and endothelial cell adhesion molecule 1 | DQLIYNLLK  NSNDPAVFK | 0.000848 | 1.734403 | |
| **6** | P20073 | ANXA7 | Annexin A7 | ANHEEVLAAGK | 0.001706 | 3.083728 | |
| **7** | P00491 | PNP | Purine nucleoside phosphorylase | VGVNGFGR | 0.001717 | 8.214402 | |
| **8** | P63000 | RAC1 | Rac family small GTPase 1 | YEFGIFNQK  YLECSALTQR | 0.00228 | 35.10094 | |
| **9** | Q9Y5Y7 | LYVE1 | Lymphatic vessel endothelial hyaluronan receptor 1 | LLNVGFQEALK  ANQQLNFTEAK | 0.00274 | 1.969095 | |
| **10** | P62937 | PPIA | Peptidylprolyl isomerase A | IYDSGTYK  FEDENFILK | 0.003364 | 5.363798 | |
| **11** | Q01813 | PFKP | Phosphofructokinase, platelet | PAANFDAIR  AACNLLQR | 0.003473 | 16.97704 | |
| **12** | P15291 | B4GALT1 | Beta-1,4-galactosyltransferase 1 | LCTSATESEVAR | 0.003821 | 2.855998 | |
| **13** | P00338 | LDHA | Lactate dehydrogenase A | ENFQNWLK  SADTLWGIQK | 0.005095 | 3.585913 | |
| **14** | O75083 | WDR1 | WD repeat domain 1 | TLPAAAFR  VINSVDIK | 0.011054 | 6.01548 | |
| **15** | P61224 | RAP1B | Member of RAS oncogene family | VVGAQSLK | 0.011257 | 8.277189 | |
| **16** | B1AK88 | CAPZB | Capping actin protein of muscle Z-line subunit β | ESCDSALR  STLNEIYFGK | 0.012655 | 5.851881 | |
| **17** | P40197 | GP5 | Glycoprotein V platelet | VVVCDNGTGFVK  LPNLSSLTLSR | 0.013058 | 4.151566 | |
| **18** | P11216 | PYGB | Glycogen phosphorylase B | YDPTIEDSYR  FSAFLEK | 0.013532 | 4.445477 | |
| **19** | P61160 | ACTR2 | Actin-Related protein 2 | TEWLDGK | 0.015716 | 21.79218 | |
| **20** | P52907 | CAPZA1 | Capping actin protein of muscle Z-line subunit alpha 1 | TVFDEAIR  LLLNNDNLLR | 0.017213 | 5.714081 | |
| **21** | O00194 | RAB27B | Member RAS oncogene family | DVNAAIAAIK  SLTTAFFR | 0.022226 | 9.810332 | |
| **22** | Q5R345 | SELP | Selectin P | NVIFQPVAELK  TWTWVGTK | 0.024216 | 3.143465 | |
| **23** | P68366 | TUBA4A | Selectin P | QVIELAGK  EIIDPVLDR | 0.027705 | 6.504806 | |
| **24** | Q5JXI8 | FHL1 | Four and a half LIM domains 1 | NPITGFGK  DCFTCSNCK | 0.028076 | 12.71164 | |
| **25** | P37802 | TAGLN2 | Transgelin 2 | LEGPNNVECTTSGR NFSDNQLQEGK | 0.03183 | 4.762219 | |
| **26** | O15144 | ARPC2 | Actin related protein 2/3 complex subunit 2 | LPIGDVATQYFADR | 0.033255 | 8.944047 | |
| **27** | Q15063 | POSTN | Periostin | NGVGVLIWK | 0.035983 | 11.00464 |  |
